# MicroRNA398: A Master Regulator of Plant Development and Stress Responses

**DOI:** 10.3390/ijms231810803

**Published:** 2022-09-16

**Authors:** Jing Li, Qiaoqiao Song, Zhi-Fang Zuo, Lin Liu

**Affiliations:** Guangdong Provincial Key Laboratory for Plant Epigenetics, Longhua Bioindustry and Innovation Research Institute, College of Life Sciences and Oceanography, Shenzhen University, Shenzhen 518060, China

**Keywords:** microRNA398, target gene, plant development, stress responses

## Abstract

MicroRNAs (miRNAs) play crucial roles in plant development and stress responses, and a growing number of studies suggest that miRNAs are promising targets for crop improvement because they participate in the regulation of diverse, important agronomic traits. MicroRNA398 (miR398) is a conserved miRNA in plants and has been shown to control multiple stress responses and plant growth in a variety of species. There are many studies on the stress response and developmental regulation of miR398. To systematically understand its function, it is necessary to summarize the evolution and functional roles of miR398 and its target genes. In this review, we analyze the evolution of miR398 in plants and outline its involvement in abiotic and biotic stress responses, in growth and development and in model and non-model plants. We summarize recent functional analyses, highlighting the role of miR398 as a master regulator that coordinates growth and diverse responses to environmental factors. We also discuss the potential for fine-tuning miR398 to achieve the goal of simultaneously improving plant growth and stress tolerance.

## 1. Introduction

Plants are continuously exposed to various biotic and abiotic stresses during their growth and development. In light of the challenges imposed by the global climate crisis, the need for improved plant varieties is increasing and urgent [1]. For the genetic improvement of crop plants, an in-depth understanding of regulators of growth, development and stress responses is key to improving agronomic traits [2]. microRNAs (miRNAs) are important post-transcriptional regulators of gene expression and play pivotal roles in various biological processes. The verified targets of miRNAs mostly encode transcription factors (TFs) and enzymes in signaling pathways, indicating that miRNAs play vital roles at the core of gene regulatory networks [3]. microRNA398 (miR398) is a highly conserved miRNA which is widespread in angiosperms [4]. As one of the earliest miRNAs cloned from Arabidopsis and rice [5,6], extensive studies of the mechanism of transcriptional regulation, biological function and environmental stress responses of miR398 have been carried out; the function of miR398 in plant stress responses has been reviewed by Zhu et al. [7]. Due to the rapid progress in the study of miR398 in different plant species and the deeper understanding of the functional mechanisms of miR398 gained in recent studies, we present a new synthesis of how the miR398 family is regulated, its function in different plant species, its responses under different stress conditions and the exciting potential for applications in crop improvement.

## 2. Evolutionary Analysis of MiR398

MiR398 is an ancient miRNA whose evolution predates the divergence of gymnosperms and angiosperms 305 million years ago [4]. Thus, it is present in gymnosperms, such as European spruce (*Picea abies*), which has two members of the miR398 family: pab-miR398a and pab-miR398b, while pteridophytes (e.g., *Marsilea quadrifolia*), lycopods (e.g., *Selaginella moellendorffii*) and the moss, *Physcomitrella patens*, have no miR398 [4,8]. MiR398 is widespread and conserved in angiosperms, and in all land plants, miR398 has a length of 21 nucleotides (nt), starting with a uracil at the 5′ end [4]. The ancient nature of miR398 suggests that it has been involved in the regulation of gene expression and plant developmental processes since the earliest stages of plant evolution.

**Figure 1:**
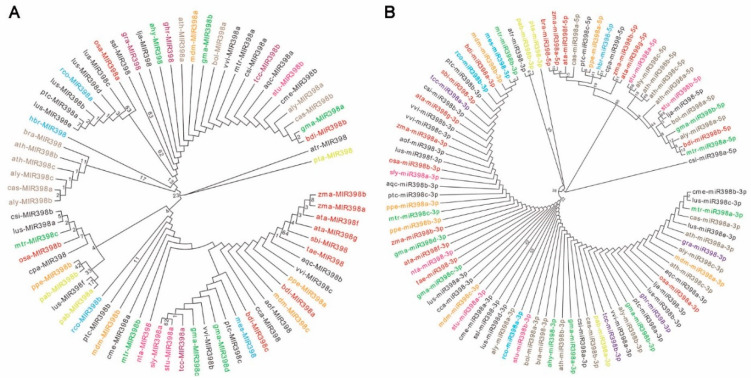
Phylogenetic analysis and sequence alignment of microRNA398 (miR398) precursor and mature sequences. (**A**) Phylogenetic analysis of the 73 miR398 precursors from 39 plant species, ranging from gymnosperms to eudicots. (**B**) Phylogenetic relationships among 93 mature miR398 sequences. Letters of the same color represent the same species. The dendrogram was constructed using MEGA7 software by the maximum-likelihood method with 1000 bootstraps. *Pinus taeda* is at the root of the evolutionary tree. ahy, *Arachis hypogaea*; aly, *Arabidopsis lyrata*; aof, *Asparagus officinalis*; aqc, *Aquilegia caerulea*; ata, *Aegilops tauschii*; ath, *Arabidopsis thaliana*; atr, *Amborella trichopoda*; bdi, *Brachypodium distachyon*; bol, *Brassica oleracea*; bra, *Brassica rapa*; cas, *Camelina sativa*; cca, *Cynara cardunculus*; cme, *Cucumis melo*; cpa, *Carica papaya*; csi, *Citrus sinensis*; ghr, *Gossypium hirsutum*; gma, *Glycine max*; gra, *Gossypium raimondii*; hbr, *Hevea brasiliensis*; lja, *Lotus japonicus*; lus, *Linum usitatissimum*; mdm, *Malus domestica*; mes, *Manihot esculenta*; mtr, *Medicago truncatula*; nta, *Nicotiana tabacum*; osa, *Oryza sativa*; pab, *Picea abies*; ppe, *Prunus persica*; pta, *Pinus taeda*; ptc, *Populus trichocarpa*; rco, *Ricinus communis*; sbi, *Sorghum bicolor*; ssl, *Salvia sclarea*; sly, *Solanum lycopersicum*; stu, *Solanum tuberosum*; tae, *Triticum aestivum*; tcc, *Theobroma cacao*; vvi, *Vitis vinifera*; zma, *Zea mays*.

To explore the evolutionary history of miR398, we downloaded all known precursor and mature sequences of miR398 family members from the miRNA database, miRBase (version 22.1) (http://www.mirbase.org/), and constructed separate phylogenetic trees of the precursor sequences and the mature sequences (Figure 1). Seventy-three miR398 precursors are listed in Table S1, and nighty-three sequences of miR398-5p/-3p, which are produced from both arms of miRNA precursors, are listed in Table S2. The precursors of the miR398 family members are relatively scattered and are distributed on different subclades (Figure 1A). However, in these species, the mature sequences of miR398-3p are highly conserved, while most mature miR398-5p sequences are quite species-specific. The conservation of miR398-3p among species may be due to it being a functional sequence subject to evolutionary selection, whereas miR398-5p is under less evolutionary pressure as an miRNA*. Clearly, the mature miR398 sequences divided into two branches, i.e., miR398-5p and miR398-3p form two distinct clades with strong bootstrap support (Figure 1B).

## 3. MiR398 Has Both Conserved and Species-Specific Target Genes

In plants, miRNAs function by binding with complementary target mRNAs. As a conserved miRNA, miR398 has been found to target genes that are conserved in different species. It was first identified in Arabidopsis and rice, and its targets were computationally predicted by the base-pairing principle [5,6,9]. As the earliest identified target genes of miR398, *Cu/Zn superoxide dismutase1* (*CSD1*) and *CSD2* are conserved among species and are responsible for scavenging reactive oxygen species (ROS) [10,11]. The miR398-*CSD* regulation module is conserved among seed plants, while the moss (*P. patens*) has no miR398, and *PpCSD1* is targeted by miR1073 instead [12]. *MITOCHONDRIAL CYTOCHROME OXIDASE SUBUNIT V* (*COX5b*) was also predicted as a target gene of miR398 due to the low number of mismatches between miR398 and *COX5b* mRNA [6]. *COPPER CHAPERONE FOR SUPEROXIDE DISMUTASE* (*CCS1*) was identified as a target later, due to the presence of 4.5 mismatches with miR398, which is more than the cut-off of 3 or 3.5 mismatches in computational target prediction methods in Arabidopsis [13]. *CCS1* is a conserved target for miR398 in rice and encodes a copper chaperone for superoxide dismutases (SODs), which delivers copper to CSD1 and CSD2 [14]. In addition to these four conserved target genes, several species-specific miR398 target genes have been identified. *BLUE COPPER-BINDING PROTEIN* (*BCBP*) is a target gene that was not discovered by computational prediction due to a bulge of 6 nt opposite to the 5′ region of miR398 [15]. Reducing the bulge did not affect miR398-mediated regulation, and completely removing it increased the slicing efficiency; this 6 nt bulge in the miR398-*BCBP* interaction region seems to be rather unique in Arabidopsis [15]. Unlike the Arabidopsis miR398/*BCBP* pairing, *BCBP* is targeted by conventional base-pairing with miR398 in *N. benthamiana*, and the cleavage was confirmed by 5′-RACE [16]. *Nodulin 19* (*Nod19*), a novel target of miR398 in common bean (*P. vulgaris*), is an ortholog of *MtN19* in *M. truncatula*, although *MtN19-like* transcripts have only been proposed as miR398 targets in soybean [17,18]. *ASCORBATE PEROXIDASE6* (*APX6*) is a potential target of miR398 in Arabidopsis that regulates age-dependent leaf senescence [19]. Three *AGAMOUS-LIKE* (*AGL*) TF genes (-*AGL51*, *AGL52* and *AGL78*), which control ovule development, are newly identified targets of miR398 in Arabidopsis [20]. It appears that the miR398 conservatively targets *CSD*/*CCS* genes to control ROS scavenging processes across species, whereas miR398 regulates developmental processes through species-specific targets.

## 4. Multi-Layered Functional Regulation of MiR398

The expression of miR398 itself is regulated at the transcriptional and post-transcriptional levels. There are multiple cis-elements in the promoter region of *MIR398* genes, examples of which are the (ABA)-responsive element (ABRE), the anaerobic induction element (ARE) and the W-box, which is essential for the binding of WRKY TFs, implying that *MIR398* genes are regulated by stress signals [21]. The W-box is involved in cold-related gene regulation, and, therefore, cold stress-regulated *MIR398* expression may be mediated through this element [22]. ABREs are likely involved in the ABA responses of *MIR398* in Arabidopsis and poplars [23], and AREs may regulate the *MIR398* flood stress responses in Arabidopsis. The expression of *MIR398* is induced by copper deficiency [24], and this response is mediated by SQUAMOSA promoter binding protein-like7 (SPL7) TF, which binds to another motif, GTAC, in the *MIR398* promoter region [25]. Like other miRNAs, miR398 precursors are transcribed from *MIR398* gene loci. Mature miR398 derives from the processing of longer primary miRNA transcripts that adopt hairpin-like structures and are then loaded into ARGONAUTE (mainly AGO1 and AGO10) proteins to exert their function. The regulation of miR398 biogenesis is an important regulatory modality for miR398 function. Environmental pollutant phenanthrene treatment impedes the processing of primary transcripts of miR398 (pri-miR398) to precursor miR398 (pre-miR398) and reduces the abundance of mature miR398, thereby alleviating the toxicity of polycyclic aromatic hydrocarbons (PAHs) to crops [26].

Natural antisense transcripts (NATs), a class of RNAs containing sequences complementary to sense mRNAs, repress the processing of pri-miR398, thus attenuating plant thermotolerance [27]. Long non-coding RNAs (lncRNAs) can also interfere with miR398-mediated regulation by mimicking its targets or functioning as miRNA baits/sponges in a sequence-dependent manner. In winter wheat (*T. aestivum*), three lncRNAs, lncR9A, lncR117 and lncR616, interact with miR398 and up-regulate the expression of *CSD1* indirectly by competitively binding to miR398, thereby affecting the resistance of the plant to cold stress [28]. In addition, spatiotemporally regulated miR398 biogenesis is a novel regulatory layer of gene expression in ovule development [29]. Arabidopsis *MIR398c-OE* and miR398 target gene mutant *agl78* exhibit similar abnormal female gametophyte development and a low seed-set phenotype due to the abnormal spatiotemporal expression of miR398 [20]. Pri-miR398c is transcribed in the female gametophyte and is then translocated to surrounding sporophytic tissues, where it is processed to mature miR398. AGO10 is expressed in the chalaza and sequesters miR398 there. Decreased AGO10 expression results in the ectopic accumulation of miR398 in the female gametophyte and the inhibition of *AGL51/52/78* expression, leading to compromised female gametophyte development [20].

**Figure 2:**
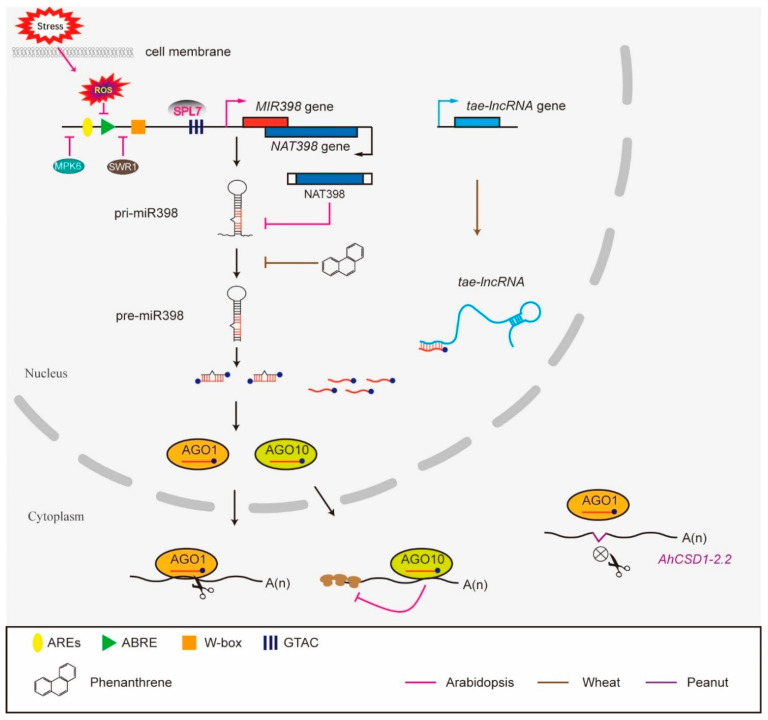
A model of the multi-layered functional regulation of miR398 in plants. (1) Stress- induced ROS signals repress the expression of miR398 via unknown factors that bind *cis*-elements in the promoter region of *MIR398*. In Arabidopsis, SPL7 directly binds to GTAC motifs in the *MIR398* promoter region and induces its expression, thereby regulating Cu distribution, while MPK6 and SWR1 repress *MIR398* expression to control ovule development (pink lines). (2) *NAT398* genes are transcribed from opposing DNA strands at the same *MIR398* locus and act as *cis*-NATs to repress miR398 biogenesis in Arabidopsis (pink line). (3) The environmental pollutant phenanthrene impedes the conversion process from pri-miR398 to pre-miR398 in wheat roots (brown line). (4) lncRNAs interact with miR398 and function as miR398 baits/sponges in wheat (brown line). (5) Some conserved targets such as *AhCSD1-2.2* in peanut bypass the regulation of miR398 by introducing mutations in the recognition site (purple line). (6) AGO10 is involved in miR398-mediated translational repression.

Secondly, the target genes of miR398 may bypass regulation under certain conditions. Mature miR398 recognizes target genes by base pairing and guides the AGO1-mediated cleavage of target mRNAs precisely between the 10th and 11th nucleotides from the 5′ end of the miRNA. Although peanut *CSD1* isoforms (*AhCSD1-1*, *AhCSD1-2.1* and *AhCSD1-2.2*) all potentially have binding sites for miR398, the binding site is eliminated in *AhCSD1-2.2* due to alternative splicing, thereby bypassing miR398-mediated suppression during drought stress [30]. This elimination of miR398 binding sites by alternative splicing and the subsequent avoidance of miR398-mediated regulation may provide a protective mechanism during peanut stress responses. In Figure 2, we summarize the multi-layered functional regulation of miR398 in plants.

## 5. The Mode of Action of MiR398 on Target Genes

MiR398 is one of the few plant miRNAs that trigger translational repression [13,31,32,33]. Many miRNAs recognize coding regions of target genes in plants, while most animal miRNA binding sites are located in 3′ untranslated regions (3′ UTRs) [13,34]. Whether the degree of complementarity or the location of the target sites affects the mode of action of miRNAs continues to be subject to debate. In the case of miR398, there is near-perfect complementarity in the coding regions of *CSD2* and *CCS1*, and it can repress mRNA expression at the translation level, suggesting that neither the degree of complementarity nor the location of target sites is a determinant of plant translational repression [13,31]. Actually, at least in vitro, plant miRNAs need almost perfect complementarity for their translational repression activity [35]. The reported miR398 target gene binding sites are either located in the 5′ UTR (*CSD1*, *COX5b*, *BCBP*) or in the coding sequence (*CSD2*, *CCS1*, *Nod19*, *APX6*), and 5′-RACE has confirmed that the cleavage by miR398 of these targets expects *APX6* [13,15,17,19].

It has been reported that, although *CSD1* and *CSD2* mRNAs accumulated in miR398-resistant plants, i.e., *rCSD1* and *rCSD2* plants (where five mutations have been introduced into the miR398 binding sites of *CSD1* and *CSD2*), the CSD1 and CSD2 protein levels remained unchanged, leading to the conclusion that miR398 was able to repress the translation of the mutated *CSD1* or *CSD2* mRNAs [31]. However, in transgenic plants expressing an miR398-resistant version of *CCS1* mRNA, both *CCS1* mRNA and protein accumulate [13]. As a chaperone for CSD1 and CSD2, CCS1 is necessary for the formation of their apolipoprotein forms, and both CSD1 and CSD2 activities are nearly absent in a null *ccs1*-knockout mutant [36]. In *rCSD1* and *rCSD2* plants, miR398 still triggers the post-transcriptional regulation of *CCS1* to keep its levels low, and this may also explain why *CSD1* or *CSD2* mRNAs accumulate but not the corresponding proteins [14]. In *ago10* mutants (*zll*), both CSD2 and CCS1 proteins accumulate without any alteration of their mRNA levels. As AGO10 is involved in miR398-directed post-transcriptional regulation, it can be concluded that miR398 acts through the translational repression and cleavage of at least *CSD2* and *CCS1* [13,31]. Whether miR398 uses both modes of target gene regulation or whether one mode is preferred over the other remains unclear.

## 6. Role of MiR398 and Its Targets in Abiotic Stress Responses

Crops and other plants in the field are subject to a combination of different abiotic stresses. The tolerance of plants to different stress combinations is an important area of research. Here, we summarize recent progress in our understanding of miR398 responses to abiotic stress in various plant species.

### 6.1. Oxidative Stress

The miR398-*CSD* module is conserved across seed plant species, and its function was first identified in the adaptation of plants to oxidative stress [11]. High concentrations of ROS, including superoxide (O_2_^•−^), hydrogen peroxide (H_2_O_2_) and hydroxyl radicals (OH^•^), can oxidatively damage biomolecules (such as lipid proteins, nucleic acids, etc.). Environmental stress caused by heavy metals, high levels of light, salinity, drought, extreme temperature, air pollutants, ultraviolet-B (UV-B) radiation, pesticides and pathogen infections leads to a rapid, excessive accumulation of ROS in plant cells, and excess ROS induce oxidative stress [37,38].

SODs play a vital role in converting superoxide to H_2_O_2_ and molecular oxygen and thus reduce oxidative stress in plant cells. As two members of SODs, CSD1 and CSD2 function in the ROS scavenging system and protect plants from oxidative stress. The expression of both CSD1 and CSD2 is induced under oxidative stress conditions due to the down-regulation of miR398 [11]. In different species and under different environmental conditions that result in oxidative stress, the expression patterns and functions of miR398 and its target genes vary (Table 1) and will be discussed in detail below.

### 6.2. Heavy-Metal, Environmental-Pollutant, High-Light, Ozone and UV Stress

Heavy metals, such as Cu^2+^, Fe^3+^ and Ni^2+^, have the potential to generate hydroxyl radicals; miR398 is down-regulated in Arabidopsis, grapevine, castor bean (*R. communis*) and hickory (*C. cathayensis*) plants exposed to heavy metals. Correspondingly, the expression levels of the target genes, *CSD1* and *CSD2*, are up-regulated in these species [11,39,40,41]. The overexpression of these miR398 targets in grapevine—*VvCSD1* and *VvCSD2*—enhances ROS scavenging systems and protects plants from metal-induced oxidative stress in tobacco (*N. tabacum*) [40]. Heavy metals cause land contamination, which adversely affects plant growth, while sulfur dioxide (SO_2_) and phenanthrene are also common pollutants that can produce oxidative toxicity in plants. SO_2_ and phenanthrene repress the expression of miR398 in Arabidopsis and wheat, and negative correlations between the levels of miR398 and its target mRNAs (*CSD1* and *CSD2*) are observed [26,42]. High-light levels cause chloroplast damage, produce ROS and significantly repress miR398 expression in Arabidopsis and rice; this stimulates the expression of *CSD1* and *CSD2*, thereby helping plants tolerate oxidative stress [11,43]. Ozone fumigation may result in another type of oxidative stress response which reduces miR398 levels and is accompanied by the up-regulation of *CSD1* but not *CSD2* [44]. miR398 expression is induced by UV-B radiation in Arabidopsis and *Populus tremula* plantlets [45,46]. It is interesting that miR398 is up-regulated by UV-B, which is contrary to its response to other types of oxidative stress. This indicates that there may be distinct machinery involved in the response to UV [46].

### 6.3. Salinity and Drought Stress

MiR398 is significantly down-regulated following NaCl treatment in Arabidopsis, cotton (*G. hirsutum*), wheat *(T. aestivum*) and tomato [21,23,47,48]. The transgenic tomato lines with sly-miR398b overexpression show increased salt sensitivity, probably due to reduced activities of SODs, ascorbate peroxidase (APX) and catalase (CAT) [49]. In other species, miR398 may have different response modes to salt stress. In poplar, miR398 expression is induced at an early stage of salt treatment and is then suppressed 48 h later, while the expression of its target gene *CSD1* shows an inverse correlation. In contrast, in Arabidopsis, miR398 is steadily and unidirectionally suppressed under similar conditions [23]. Even in the same plant, different tissues may have contrasting responses to salt stress: in response to salt stress, miR398 is down-regulated in sweet potato roots but up-regulated in leaves, suggesting that the miR398 regulation of abiotic stress responses is dependent on the plant developmental context [50].

Similarly, miR398 shows different responses to drought stress in different species. miR398 is down-regulated by drought in tomato [48] and cotton [51] and up-regulated in wild emmer wheat [52] and peanut [30], but it is unaltered in switchgrass [53]. miR398 overexpression lines are sensitive to drought stress in rice, indicating the negative regulation of miR398 in wild-type rice subjected to drought [43].

### 6.4. Water Deficit and Flood Stress

Water-related hazards such as water deficiency and flood are increasing because of climate change, and both cause stress in plants. In *M. truncatula*, miR398a and miR398b are up-regulated in both shoots and roots under water-deficit conditions, corresponding with the down-regulation of the miR398a target, *COX5b* (*TC123882*). As the COX5b protein forms part of the electron transport chain in mitochondria, these results highlight the involvement of miR398 in the regulation of mitochondrial respiration in response to water deprivation in *M. truncatula* [54]. In contrast, in pea (*P. sativum*) and common bean (*P. vulgaris*), miR398 accumulation is reduced, whereas *CSD1* expression is enhanced upon water deficit [55,56]. In addition to water deficit, flooding (also known as waterlogging or submergence) is another major abiotic stress that restricts plant growth. Too much water causes the submergence of plants, which induces hypoxia: miR398 is down-regulated under these conditions in Arabidopsis, suggesting a role for miR398 in low-oxygen signaling [57].

### 6.5. Thermal Stress

Extreme temperatures induce ROS, and it appears that miR398 has different regulatory mechanisms for cold and heat stresses. Low temperature is a major abiotic stress affecting crop production in high-latitude areas, and cold stress suppresses miR398 expression in various species. MiR398 was first reported to be down-regulated during cold stress in Arabidopsis [6]. Similarly, in *Chrysanthemum dichrum* the expression level of miR398 is reduced upon freezing treatment, while the targets *CSD1* and *CSD2* show correspondingly elevated expression levels [58]. In winter turnip rape (*Brassica rapa*), which is also a dicotyledonous plant, miR398 is up-regulated in leaves under cold stress conditions [59], suggesting that the response of miR398 to cold stress is species-specific. In the monocot wheat (*T. aestivum*), the expression of miR398 also decreases in response to low temperatures, and, correspondingly, its target *CSD1* shows the opposite expression pattern [21,28]. Cold stress may lead to cellular dehydration and membrane damage, thereby inducing ROS; the inhibition of miR398 and the up-regulation of *CSD* may be involved in ROS scavenging during cold adaptation.

MiR398 is an ambient temperature-responsive miRNA [60]; it responds not only to cold stress but also to heat stress. Heat stress rapidly induces miR398 expression and reduces transcripts of its target genes *CSD1*, *CSD2* and *CCS1* in Arabidopsis. Although *CSD* and *CCS1* act as antioxidants which enhance plant resistance to abiotic stresses, mutations in *CSD1*, *CSD2* and *CCS1* improve heat-tolerance in Arabidopsis. In contrast, transgenic plants that overexpress miR398-resistant forms of *CSD1*, *CSD2* or *CCS1* are more sensitive to heat stress due to reduced activities of heat-stress TFs and heat-shock proteins [61,62]. In monocot maize, miR398 is up-regulated, with a similar response pattern to that of the dicotyledonous Arabidopsis [61]. In Chinese cabbage (*B. rapa ssp. chinensis*), which also belongs to the Brassicaceae family, bra-miR398a and bra-miR398b are down-regulated in response to heat stress, and their target gene *BracCSD1* is up-regulated correspondingly [63]. Heat stress can induce miR398 to different levels depending on species or tissue. Thus, miR398 is only slightly down-regulated under heat stress conditions in *Populus tomentosa* and switchgrass [53,64], and in *Helianthus annuus*, miR398 is up-regulated in leaves but down-regulated in roots [65].

### 6.6. MiR398 in Nutrient Homeostasis

MiRNAs are seen as emerging targets for biotechnology-based biofortification programs and can be utilized to combat micronutrient malnutrition [66]. MiR398 plays a key role in plant responses to imbalances of major nutrients, including copper (Cu), zinc (Zn), phosphorus (P) and nitrogen (N).

The transition element Cu is important for photosynthetic and respiratory electron transport, oxidative stress protection, cell wall metabolism and ethylene perception in plants, but it is toxic when present in excess [67]. Plastocyanin (PC), Cu/Zn SODs and cytochrome c oxidase (COX) are major copper proteins in plant cells, and PC is essential for electron transfer: mutants with insertions in PC genes are seedling-lethal, while Cu/Zn SODs are relatively dispensable [24]. Based on the metal cofactors the enzyme binds, SODs can be classified into three types: Cu/Zn-SOD, Mn-SOD and Fe-SOD. In a situation of Cu deficiency, in order to preserve Cu for more important life processes, the Cu-responsive TF SPL7 binds to GTAC motifs in the *MIR398* promoter region and induces its expression, as mentioned previously. Moreover, miR398 directs the degradation of *Cu/Zn-SOD*, *CCS1* and *COX5b-1* mRNAs, allowing for the efficient delivery of Cu from CSDs to PC; Cu/Zn-SODs activities are then replaced by Fe-SOD (FSD1) [24,25,68]. Since CCS1 supplies Cu to CSDs, the miR398-dependent down-regulation of CCS1 further promotes Cu release from CSDs under Cu-deficient conditions [69]. This mechanism allows plants to save Cu for the most essential functions when the metal is in limited supply.

Zn is an essential micronutrient for plant growth and development. Cu/Zn SODs not only play a role in the regulation of Cu homeostasis but also respond to Zn deficiency in maize [70]. CSDs participate in Zn delivery, while Zn depletion depresses CSD function by the up-regulation of miR398 in Sorghum bicolor, suggesting that miR398 is an important regulator of the response to Zn deficiency in plants [71].

Inorganic phosphate (Pi) and N deficiency are known to be limiting factors for plant growth and agricultural productivity. Pri-miR398a and mature miR398 are down-regulated during P starvation and N limitation in Arabidopsis [72]. Pi starvation induces miR398 accumulation in tomato, *N. benthamiana* and alfalfa (*Medicago sativa*), implying a different Pi deficiency-response mechanism between Arabidopsis and these crops [16,73,74]. However, as in Arabidopsis, miR398 is down-regulated under nitrate-limiting conditions in maize and potato [75,76].

**Table 1:** MiR398 responses to abiotic stresses in diverse plant species.

Abiotic Stress	Species	MiR398	Target Genes	References
Salt	Ath	↓	*CSD1*↑, *CSD2*–	[23]
	Pte	↑&↓	*CSD1*↓&↑	[23]
	Ghi	↓	nd	[47]
	Tae	↓	nd	[21]
	Sly	↓	*CSD1*↑	[48,49]
	Iba	root↓; leaves↑	nd	[50]
Drought	Osa	↓	*CSD1*↑, *CSD2*↑	[43]
	Ttu	↑	nd	[52]
	Sly	↓	nd	[48]
	Ghi	↓	nd	[51]
	Pvi	–	nd	[53]
	Ahy	↑	*CSD1*↓, *CSD2*↓	[30]
Water deficit	Mtr	↑	*COX5b*↓	[54]
	Psa	↓	*CSD1*↑, *COX5b*–	[55]
	Pvu	↓	*CSD1*↑	[56]
Flooding	Ath	↓	nd	[57]
Cold	Ath	↓	nd	[6]
	Cdi	↓	*CSD1*↑, *CSD2*↑	[58]
	Tae	↓	*CSD1*↑	[21,28]
	Bra		nd	[59]
Heat	Ath	↑	*CSD1*↓, *CSD2*↓, *CCS1*↓, *COX5b*–	[62]
	Zma	↑	nd	[62]
	Pto	↓	nd	[64]
	Bra	↓	*CSD1*↑	[63]
	Han	root↓; leaves↑	nd	[65]
	Pvi	↓	nd	[53]
High light	Ath	↓	*CSD1*↑, *CSD2*↑	[11]
	Osa	↓	*CSD1*↑, *CSD2*↑	[43]
Heavy metal	Ath	↓	*CSD1*↑, *CSD2*↑	[11]
	Vvi	↓	*CSD1*↑, *CSD2*↑	[40]
	Cca	↓	*CSD1*↑, *CSD2*↑	[41]
Methyl viologen	Ath	↓	*CSD1*↑, *CSD2*↑	[11]
Ozone	Ath	↓	*CSD1*↑, *CSD2*–	[44]
UV	Ath	↑	nd	[45]
	Pte	↑	nd	[46]
SO_2_	Ath	↓	*CSD1*↑, *CSD2*	[42]
Phenanthrene	Tae	↓	*CSD1*↑, *CSD2*↑	[26]
Nickel	Rco	↓	*Cu-Zn/SOD*↑	[39]

Abbreviations: Ath, *Arabidopsis thaliana*; Ahy, *Arachis hypogaea*; Bra, *Brassica rapa*; Cca, *Carya cathayensis*; Cdi, *Chrysanthemum dichrum*; Ghi, *Gossypium hirsutum*; Han, *Helianthus annuus*; Iba, *Ipomoea batatas*; Mtr, *Medicago truncatula*; Pvi, *Panicum virgatum*; Pvu, *Phaseolus vulgaris*; Psa, *Pisum sativum*; Pte, *Populus tremula*; Pto, *Populus tomentosa*; Rco, *Ricinus communis*; Sly, *Solanum lycopersicum*; Tae, *Triticum aestivum*; Ttu, *Triticum turgidum*; Osa, *Oryza sativa*; Vvi, *Vitis vinifera*; Zma, *Zea mays*; ↑, up-regulated; ↓&↑, initially down-regulated then up-regulated; ↓, down-regulated; –, no change; nd, not determined.

## 7. Role of MiR398 and Its Targets in Biotic Stress Responses

The expression level of miR398 is also regulated by various biotic stresses, including bacterial, fungal and viral infection, and the miR398-*CSD* module is involved in these disease resistance responses. The responses of miR398 to biotic stresses in diverse plant species are listed in Table 2.

The model bacterial pathogen *Pseudomonas syringae* pv. tomato DC3000 infiltrates Arabidopsis leaves, which then exhibit the down-regulation of miR398 expression. *CSD1*—but not *CSD2*—mRNA levels are up-regulated in response to this biotic stress [44]. The overexpression of miR398b enhances the susceptibility of Arabidopsis to DC3000 and flg22, a conserved peptide derived from *P. syringae* flagellin, indicating that miR398b negatively regulates Arabidopsis disease resistance [77]. Flg22 suppresses miR398b accumulation, and, consistent with this, the expression of the miR398 target genes *COX5b-1*, *CSD1* and *CSD2* is increased [77]. The down-regulation of miR398 is also observed in citrus plants infected with harmful bacteria of the genus *Candidatus Liberibacter* [78].

In contrast to its negative role in defense against bacteria in Arabidopsis, miR398b is reported to positively regulate rice immunity against the blast fungus *Magnaporthe oryzae* [79]. Transgenic rice plants overexpressing miR398b display enhanced resistance to M. oryzae, which is associated with the reduced mRNA levels of miR398b targets in rice, i.e., *CSD1*, *CSD2*, *SODX* and *CCSD* [79,80]. The underlying mechanism by which miR398b regulates rice blast resistance involves boosting total SOD activity and increasing H_2_O_2_ concentration, thereby improving disease resistance [80]. In contrast, the transient expression of common bean miR398b in *N. benthamiana* leaves results in enhanced lesions caused by the fungus, *Sclerotinia. sclerotiorum* [17]. Powdery mildew is another major disease that reduces crop yields and is usually caused by the ascomycete fungus *Blumeria graminis* f. sp. *hordei.* MiR398 is involved in the defense against powdery mildew by regulating the expression of *SOD1* in barley: the accumulation of *SOD1* enhances plant resistance [81]. Interestingly, the expression patterns of miR398 vary with different types of biotic interaction. In the roots of bread wheat (*T. aestivum*), miR398 expression increases during the early response to *Fusarium culmorum* inoculation [82]. A down-regulation of miR398b and an up-regulation of *CSD1* and the bean-specific miR398 target gene *Nod19* occur in common bean (*P. vulgaris*) leaves challenged with *S. scleortiorum* [17].

It is likely that pathogens can manipulate miR398 levels to facilitate their infection. Studies of many species have shown that virus-infected plants exhibit elevated miR398 accumulation. *Tobacco mosaic virus* (TMV) and *oilseed rape mosaic tobamovirus* induce the expression of miR398 in *Arabidopsis* [83,84]. *Tomato leaf curl virus* (TolCNV)-infected tomato [85], potato virus X (PVX)- and potato virus Y (PVY)-infected *N. benthamiana* [86], *Papaya meleira virus* (PMeV)-infected Carica papaya [87] and *potato spindle tuber viroid* (PSTVd)-infected tomato [88] show miR398 accumulation. Whether *CSD* transcript levels are altered by viral infection may depend on the host–virus combination. Thus, PVX and PVY infection results in higher *NbCSD* transcript levels in tobacco, whereas ToLCNV infection reduces *CSD1* and *CSD2* expression in tomato. *Bamboo mosaic virus* (BaMV) infection increases the level of miR398, but this up-regulation of miR398 does not have an anti-BaMV effect, instead promoting the manifestation of virus-infection symptoms by increasing the levels of ROS [89]. In *N. benthamiana*, *beet necrotic yellow vein virus* infection induces miR398, and Liu et al. speculated that miR398 enhances plant resistance against viruses by targeting *umecyanin* [90].

**Table 2:** MiR398 responses to biotic stresses in diverse plant species.

Biotic Stresses	Species	MiR398	Target Genes	References
**Bacteria**				
*Pst* DC3000	Ath	↓	*CSD1*↑, *CSD2*–	[44]
Flg22	Ath	↓	*CSD1*↑, *CSD2*↑, *COX5b*↑	[74]
Ca. L.	Csi	↓	nd	[78]
**Fungus**				
*M. oryzae*	Osa	↑	nd	[79,80]
*bgh*	Hvu	↓	*HvSOD1*↑	[81]
*S. scleortiorum*	Pvu	↓	*CSD1*↑, *NOD19*↑	[17]
*F. culmorum*	Tae	↑	nd	[82]
**Virus**				
TMV	Ath	↑	nd	[84]
ORMV	Ath	↑	nd	[83]
TolCNV	Sly	↑	*CSD1*↓, *CSD2*↓	[85]
PVX/Y	Nbe	↑	*CSD1*↑, *CSD2*↑	[86]
PMeV	Cpa	↑	nd	[87]
PSTVd	Sly	↑	nd	[88]
BaMV	Bdi	↑	*CSD2*↑	[89]
BNYVV	Nbe	↑	nd	[90]

Abbreviations: pathogens: Pst DC3000, *Pseudomonas syringae* pv. tomato, (Pst) DC3000; Ca. L., *Candidatus Liberibacter*; *M. oryzae*, *Magnaporthe oryzae*; *Bgh*, *Blumeria graminis f. sp. hordei*; *S. sclerotiorum*, *Sclerotinia scleortiorum*; *F. culmorum, Fusarium culmorum*; TMV, *Tobacco mosaic virus*; ORMV, *Oilseed rape mosaic tobamovirus*; TolCNV, *Tomato leaf curl virus*; PVX/Y, *Potato virus X/Y*; BaMV, *Bamboo mosaic virus*; BNYVV, *Beet necrotic yellow vein virus*. PMeV, *Papaya meleira virus*; PSTVd, *Potato spindle tuber viroid*; plant species: Ath, *Arabidopsis thaliana*; Bdi, *Brachypodium distachyon*; Cpa, *Carica papaya*; Csi, *Citrus sinensis*; Hvu, *Hordeum vulgare*; Nbe, *Nicotiana benthamiana*; Pvu, *Phaseolus vulgaris*; Sly, *Solanum lycopersicum*; Tae, *Triticum aestivum*; Osa, *Oryza sativa*. ↑, u—regulated; ↓, down-regulated; –, no change; nd, not determined.

## 8. Role of MiR398 in Plant Growth and Development

Both miR398 and its targets *CSD1/2* show spatial and temporal expression patterns, implying a role of miR398 and its target genes in the regulation of plant growth and development [11]. However, compared with research on the role of miR398 in stress-response regulation, there are relatively few studies on its function in growth and development.

Nevertheless, based on the reports to date, it appears that miR398 regulates growth via target genes other than the various *CSDs*. In the Arabidopsis *ccs* knockout mutant, almost all Cu/Zn SOD activity is lost; yet, these plants are phenotypically similar to the wild type under normal growth conditions [36]. MiR398 overexpression lines do not exhibit any visible growth phenotype despite a severe reduction in activity of both CSD1 and CSD2 [24]. A more detailed phenotypic characterization showed that *MIR398c* overexpression lines have reduced fertility and abnormal female gametophytes, although a mutant line with a T-DNA insertion in the *MIR398c* locus, resulting in reduced *MIR398c* expression, exhibits no obvious defects in ovule development [20]. The role of *miR398* in female gametophyte development involves *AGL51*, *AGL52* and *AGL78*, which belong to the *MADS-box* gene family. Female gametophyte development and ovule morphogenesis are regulated by the temporal and spatial control of miR398 biogenesis: mature miR398 is sequestered by AGO10 in the female gametophyte to ensure the expression of its targets *AGL51/52/78*, which are essential for female gametophyte development as well as integument growth [20]. In Arabidopsis, miR398 also regulates age-dependent leaf senescence by targeting *APX6* [19].

In crops, miR398 is reported to regulate rice growth and yield. The overexpression of miR398 can increase panicle length, grain number and grain size in rice, and miR398 suppression in transgenic short tandem target mimic (STTM398) lines shows a significant decrease in grain length, width and 1000-grain weight [91]. Transgenic lines carrying a resistant version of one of the miR398 targets, a rice *CSD2* homolog, show wild-type phenotypes for seed length, seed width and 1000-grain weight. The phenotype of yield-related traits in miR398 overexpression and STTM398 lines indicates that the manipulation of miR398 is a promising strategy that may lead to important yield improvements [91].

## 9. Conclusions and Perspectives

Regulators of plant growth, development and stress responses are important factors in plant evolution and domestication, and these factors are key targets for plant genetic improvement. Studies on the functions of these key regulators and the regulatory networks in which they participate are required for the transformation from basic research to application. miR398 works as a master regulator of plant responses to multiple environmental stresses and of growth and development. In response to stresses, the miR398-*CSD* module appears to be at the core of several gene regulatory networks: the upstream TF SPL7 regulates the expression of miR398, while the downstream CSD pathway regulates ROS-responsive genes. In growth and development, miR398 regulates growth-related target genes to achieve specific control. MiR398 integrates multiple developmental and environmental signals to fine-tune gene expression and to achieve the coordination of various growth and physiological processes. Considering its universality among angiosperm species, as well as the multiple layers of regulation in various species, miR398 makes a highly promising target for crop breeding and biotechnology. However, a deeper understanding of the underlying mechanisms of miR398-target function and regulation is required to fully utilize its biotechnological potential. Since miR398 appears to regulate growth and development through species-specific target genes, the identification of non-conserved miR398 target genes in different plant species by degradome sequencing will be a focus in future research, and more functional work is required to reveal the mechanism of action of these species-specific targets. In addition, as the evolutionarily conserved miR398 is usually encoded by multiple family members, and different members may exhibit distinct expression patterns and responses to environmental stresses, it is necessary to explore the specific functions and contributions of each family member. With the development of the CRISPR/CAS9 genome editing system, it is now possible to generate loss-of-function mutants for different miR398 family members for functional analysis. Since the exogenous transgene sequences used for genome editing are easily removed in the subsequent generation, the edited miR398 mutants may serve as valuable resources for crop improvement.

## Data Availability

Not applicable.

## Supplementary Materials

The supporting information can be downloaded at: https://www.mdpi.com/article/10.3390/ijms231810803/s1.
